# Associations of hearing loss and hearing aid use with brain pathologies at autopsy in a community‐based cohort

**DOI:** 10.1002/alz70860_107572

**Published:** 2025-12-23

**Authors:** Willa D Brenowitz, Michael L. Lee, Caitlin S Latimer, Paul K Crane, Laura E. Gibbons, Nicole M Gatto, Dennis L Barbour, Frederick J Gallun, Eric B Larson, C. Dirk Keene, Aaron R Seitz, Linda K. McEvoy

**Affiliations:** ^1^ Kaiser Permanente Center for Health Research, Portland, OR, USA; ^2^ Department of Medicine, University of Washington, Seattle, WA, USA; ^3^ University of Washington, Seattle, WA, USA; ^4^ Department of General Internal Medicine, University of Washington School of Medicine, Seattle, WA, USA; ^5^ Department of Medicine, University of Washington School of Medicine, Seattle, WA, USA; ^6^ Kaiser Permanente Washington, Seattle, WA, USA; ^7^ McKelvey School of Engineering, Washington University in St Louis, St Louis, MO, USA; ^8^ Oregon Health and Sciences Univeristy, Portland, OR, USA; ^9^ University of Washington, School of Medicine, Seattle, WA, USA; ^10^ Northeastern University, Boston, MA, USA; ^11^ Kaiser Permanente Washington Health Research Institute, Seattle, WA, USA

## Abstract

**Background:**

Hearing loss is associated with higher risk of dementia. Treatment with hearing aids may reduce this risk; however, the underlying mechanisms are unclear. Few community‐based studies have evaluated associations between hearing abilities, hearing aid use, and neuropathologic features from brain autopsy. We examined whether neuropathologic profiles differed between those with and without hearing impairment identified from medical records. We focused on neuropathologic features of Alzheimer's disease: Lewy body disease, hippocampal sclerosis, and vascular brain injury, all of which are associated with dementia.

**Method:**

We studied autopsy and clinical hearing history data of 850 participants aged ≥65 in the Adult Changes in Thought study, which began in 1994. Hearing impairment and hearing aid use were determined based on medical record review. Logistic regressions evaluated associations of impaired hearing and hearing aid use with common neuropathological findings, adjusting for sex and education, and age at death. Neuropathological findings included Alzheimer's disease neuropathologic change (ADNC) (defined by a modified score of 0‐3 based on CERAD neuritic plaque density and Braak Stages for neurofibrillary degeneration), neocortical Lewy body disease, hippocampal sclerosis, number of gross infarcts, and number of microinfarcts. Relative risks (RR) were estimated using marginal standardization.

**Result:**

About half the participants had dementia by their last clinical visit (45.4%) with a mean age at death of 88.7 years (SD: 6.7). Documented hearing loss was common (67.1%) and 36.8% were hearing aid users. Hippocampal sclerosis but not other pathologies was associated with documented hearing loss prior to death (RR: 1.13; 95% CI: 1.003, 1.28). Among 479 participants with documented hearing loss, hearing aid use was associated with lower ADNC level (RR per one unit increment in ADNC score: 0.94; 95% CI: 0.89, 0.99). There were no other significant associations between hearing aid use and other neuropathologies.

**Conclusion:**

In this community‐based sample, clinically diagnosed hearing loss was associated with hippocampal sclerosis but not other neuropathologies. Hearing aid use intriguingly was associated with lower levels of ADNC. However, future work will be needed to replicate these findings and establish the casual pathways underlying these associations.

